# Application of Patient-Reported Outcome Measurements in Clinical Trials in China

**DOI:** 10.1001/jamanetworkopen.2022.11644

**Published:** 2022-05-11

**Authors:** Hui Zhou, Mi Yao, Xiaodan Gu, Mingrui Liu, Ruifeng Zeng, Qin Li, Tingjia Chen, Wen He, Xiao Chen, Gang Yuan

**Affiliations:** 1 Phase I Clinical Trial Center, the First Affiliated Hospital, Sun Yat-sen University, Guangzhou, China; 2 Department of Pharmacy, the First Affiliated Hospital, Sun Yat-sen University, Guangzhou, China; 3 Institute of Applied Health Research, University of Birmingham, Birmingham, United Kingdom; 4 School of Pharmaceutical Sciences, Sun Yat-sen University, Guangzhou, China; 5 Department of Geriatrics, the First Affiliated Hospital, Sun Yat-sen University, Guangzhou, China; 6 Healthcare Center, the First Affiliated Hospital, Sun Yat-sen University, Guangzhou, China

## Abstract

**Importance:**

Regulatory authorities, industry peers, and international health policies have emphasized the value of assessing patient-reported outcomes (PROs) in clinical studies. Despite the increase in the number of clinical studies in the last decade in China, little is known about the extent of the use of PROs.

**Objective:**

To evaluate the application and characteristics of PRO instruments as primary and secondary outcomes in randomized clinical trials in China.

**Design, Setting, and Participants:**

A cross-sectional study of interventional clinical trials conducted in China from January 1, 2010, to December 31, 2020, was performed. Data obtained from the Chinese Clinical Trial Registry and ClinicalTrials.gov databases were evaluated.

**Main Outcomes and Measures:**

Trials were categorized according to those that (1) precisely listed PRO tools as outcomes, (2) mentioned patient subjective feelings in outcomes but did not clarify which tools were used for assessment, and (3) did not mention any PRO measurements. Data on study phase, setting, participant age, and sex were extracted from trials that considered patient feelings, along with the target diseases and names of the PRO tools.

**Results:**

Among a total of 34 033 trials, 6915 (20.3%) listed the explicit PRO instruments used and 3178 (9.3%) included PRO in their outcomes but did not include the names of the assessment tools. From more than 32 million people included in the registered trials, data on 1.5 million (4.7%) patients were scientifically collected by PRO instruments, and subjective feelings were assessed for 693 867 (2.1%) participants. Pain (16.8%), cancer (15.6%), and musculoskeletal symptoms (13.3%) were the most common conditions for which PROs were precisely collected by tools. The most common tools for PRO measurements were the visual analog scale, Short-Form 36, and Hamilton Depression Scale.

**Conclusions and Relevance:**

In this cross-sectional study, the use of PROs increased during the study period in clinical trials conducted in China. However, patient opinion appears to still be rarely measured. The application of PRO is geographically unevenly distributed. Development of PRO instruments, especially those suitable for the Chinese population, may be useful. Further expansion of PROs with respect to the scope of diseases is needed to avoid missing important data.

## Key Points

**Question:**

What is the status of the use of patient-reported outcome (PRO) tools in randomized clinical trials conducted in China?

**Findings:**

This cross-sectional study of 34 033 clinical trials conducted in China from 2010 to 2020 revealed that few used PRO instruments, representing less than 5% of the participants, mainly involving pain, cancer, and musculoskeletal disease. PROs were commonly evaluated with the visual analog scale and the Short Form 36-Item Health Survey.

**Meaning:**

These findings suggest that the application of PROs in clinical trials in China could be enhanced; much research has missed the opportunity to assess patient experience, which may lead to a lack of crucial data.

## Teaser

This cross-sectional study evaluates the use of patient-reported outcome instruments as primary and secondary outcomes in randomized clinical trials conducted in China.

## Introduction

A patient-reported outcome (PRO) constitutes information about symptom severity, disease state, and change from a previous measure that comes directly from patients.^1,2^ PROs provide evidence for evaluating the disease status or changes during treatment.^3^ Rigorously captured PRO results of trials have substantial implications for pharmaceutical labeling claims,^4^ product reimbursement,^3^ and decision-making on medicine use,^5^ and may even be factors in health care policy.^5^ PROs have been widely used to understand the perspective of patients in randomized clinical trials^5^ and clinical practice^6^ and have several advantages, including being noninvasive, patient-centered, and easily accessed.^7^

With PROs becoming an area of greater interest, an increasing number of studies have been using PROs as one of the crucial primary or secondary end points.^8,9,10,11^ PRO measurements have been used in some high-quality randomized clinical trials as alternatives to the traditional outcomes used to evaluate the efficacy of treatment.^12,13,14^ Some researchers believe that data collected using PROs would be more comprehensive than routine medical records and have better sensitivity.^8,15,16^ In particular, PRO measurements that follow guidelines^1,2,4^ for study design, data collection, and data analysis are recognized to be highly evidence-based. With the value of PROs being widely accepted, questions have been raised about the extent and scope of PRO use in studies. Some researchers believe that understanding and evaluating the application of PROs is a necessary step for further improving the quality of clinical studies. Studies on the standardization of the PRO collection process for clinical trials have been conducted.^17,18,19^ The results of those studies suggest that the application of PROs is inadequate, incomplete, or underreported.^20,21,22^ To our knowledge, there have been no comprehensive evaluations assessing the use of PROs in clinical trials in China.

China has the potential to recruit an adequate number of participants for clinical trials of any size. The number of clinical trials is also increasing as the population increases. Therefore, we reviewed the registration information of randomized clinical trials conducted in China to understand how PROs were being used to gain insight on some possible directions for future efforts.

## Methods

### Study Design

This cross-sectional study was designed to calculate the number and evaluate the characteristics of clinical trials conducted in China that have used PROs as their primary or secondary end points from January 1, 2010, to December 31, 2020. All clinical trials should be registered, and the World Health Organization regards registration of clinical trials as a scientific, ethical, and moral responsibility. Approximately 99%^23^ of the clinical trials conducted in China are registered on the 2 primary World Health Organization registries: ClinicalTrials.gov and the Chinese Clinical Trial Registry, with public disclosure. We limited our search to intervention studies in the 2 databases (eMethods in the Supplement). We further sought to identify which PRO tools were most commonly used in trials for different target diseases. Because this study did not involve clinical data or human participants, it was exempt from institutional review board approval and the requirement for informed patient consent per the Common Rule (45 CFR part 46). This study followed the Strengthening the Reporting of Observational Studies in Epidemiology (STROBE) reporting guideline.

### Data Collection Strategy

**Figure 1.**
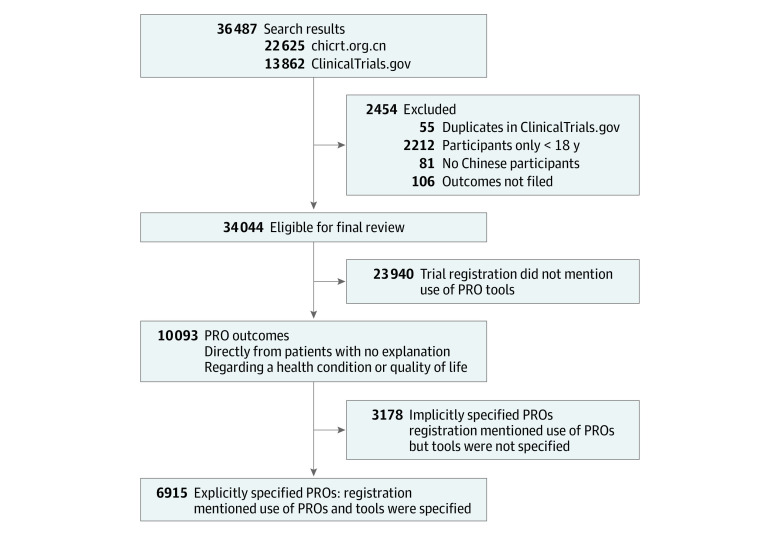
Trial Exclusion and Classification Criteria PRO indicates patient-reported outcome.

We included trials (1) for which the country of the primary sponsors or recruitment/research settings was China, (2) were interventional (randomized clinical trials), and (3) recruited participants older than 18 years (Figure 1). Trials with 2 registration identification numbers were considered duplicate and were counted only once (in the Chinese Clinical Trial Registry) to avoid deviations caused by repeated statistics. The types of information included to assess the outcomes and characteristics of the clinical trials were as follows: (1) basic information (registration number, date of registration, scientific title, countries of recruitment, and research settings), (2) key information (outcomes, target disease, and participant age and sex), and (3) feature information (primary sponsor, primary sponsor location, source of funding, and study phase).

### Data Classification

Eligible trials were categorized into 3 groups according to the outcomes reported: (1) explicitly specified PROs (trial registration mentioned the use of PRO tools and PRO tools were specified), (2) implicitly specified PROs (trial registration mentioned the use of PRO tools, but PRO tools were not specified), or (3) PROs not mentioned (trial registration did not mention the use of PRO tools). PRO instruments were categorized according to the definition of the US Food and Drug Administration guideline for industry issued in 2009^4^ as any form of measurements that come directly from the patients without any explanation from others about a health condition or quality of life.

### Statistical Analysis

Data related to the characteristics of the included studies were extracted independently by 2 of us (H.Z. and X.G.), using predesigned data extraction tables. The study phase, participant age and sex, and study setting are summarized in Table 1. Owing to the wide variation in target diseases, similar conditions were categorized in the same groups (eTable 1 in the Supplement). Based on our categorization of conditions, the PRO instruments used in each trial were summarized to calculate the most and least frequently used tools. We included in statistical analysis only items that listed the PRO tool names for quantitative analysis to know which assessment tools were used. Descriptive statistics were summarized using SPSS software, version 27.0 (IBM Corp).

**Table 1.** Characteristics of all Trials and Trials Including PROs

Characteristics	Total, No. (%)	
	Trials	PRO trials
No.	34 033	10 093
Phase		
Early stage	439 (1.3)	131 (1.3)
1	3517 (10.3)	395 (3.9)
2	2722 (8.0)	570 (5.7)
3	2846 (8.3)	811 (8.0)
4	5112 (15.0)	1306 (12.9)
Other^a^	7738 (22.7)	2677 (26.5)
Unclear	11 659 (34.3)	4203 (41.5)
Setting		
Hospital	29 927 (87.9)	8925 (88.4)
Community	151 (0.4)	73 (0.7)
Other^b^	2545 (7.5)	765 (7.6)
Unclear	1410 (4.1)	330 (3.3)
Age		
18-no limit	31 121 (91.5)	9140 (90.6)
Over 65	1168 (3.4)	475 (4.7)
Unclear	1744 (5.1)	478 (4.7)
Gender		
Male	1496 (4.4)	471 (4.7)
Female	3540 (10.4)	1067 (10.6)
Both	28 941 (85.0)	8545 (84.7)
Unclear	56 (0.1)	10 (0.1)
Regions, China		
Southwest	3099 (9.1)	1048 (10.4)
Northeast	1182 (3.5)	360 (3.6)
North	8450 (24.8)	2276 (22.6)
Northwest	1163 (3.4)	326 (3.2)
East	12 894 (37.9)	3895 (38.6)
South	4992 (14.7)	1549 (15.4)
Central	2253 (6.6)	639 (6.3)

^a^ Investigator-initiated trials, new treatment measurements, inspection technology, health service, and therapeutic devices.

^b^ Rehabilitation, nursing home, campus, centers for disease control, home, and research institute.

## Results

### Trial Characteristics

The overall characteristics of the included trials are summarized in Table 1. We identified 36 487 interventional studies conducted in China from 2010 to 2020, including 22 625 from the Chinese Clinical Trial Registry and 13 862 from ClinicalTrials.gov. We excluded 2454 trials, including 55 duplicates, 2212 clinical trials including children, and 187 trials that were not conducted in China or were considered incomplete. A total of 34 033 eligible trials were identified for analysis.

**Figure 2.**
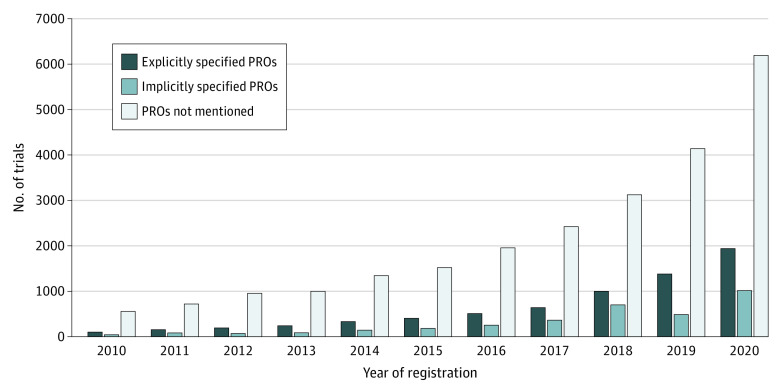
Number of Clinical Trials Analyzed PRO indicates patient-reported outcome.

Of the 32 683 284 participants in all trials, 4.7% (n = 1 520 171) were involved in trials that reported specific PRO tools and 2.1% (n = 693 867) were involved in trials that used a vague PRO description. Among the 34 033 included trials, 10 093 (29.7%) used PROs as their primary or secondary outcomes, 6915 (20.3%) listed specific PRO instruments, and 3178 (9.3%) did not name the instruments that were chosen. Most trials (23 940 [70.3%]) did not incorporate any PRO measurements (Figure 2).

More than 90% of the studies had no limit on participant age (we excluded all trials in children). The proportion of older participants (age >65 years) was higher in trials involving PRO than in those that did not. The number of clinical trials including women was 2 times higher than those including men among all trials that included PRO measurements.

Of 34 033 clinical trials, phase 4 trials (5112 [15.0%]) were the most common, followed by phase 1 (3517 [10.3%]), phase 3 (2846 [8.3%]), and phase 2 (2722 [8.0%]). Of 10 093 PRO-related trials, phase 4 trials were again the most common (1306 [12.9%]), followed by phase 3 (811 [8.0%]), with few such measures applied in phase 2 (570 [5.7%]) and phase 1 (395 [3.9%]) trials (Table 1).

Nearly 90% of the trials were conducted in hospitals, and less than 1% were performed in primary care settings. Most primary sponsors were located in eastern China, followed by northern and southern China; the other regions, including southwestern, central, northwestern, and northeastern China, accounted for less than 0.1% of the trial locations (Table 1). There were similar findings when considering only the PRO-related studies, with more than 70% of primary sponsors coming from the eastern, northern, and southern areas of China; fewer than 30% of sponsors and research teams were from the southwestern, central, northeastern, and northwestern areas of China (Table 1). There were substantial differences in the proportion of PRO trials among the different provinces. The percentage PROs in Chinese provinces is shown in the eFigure in the Supplement.

### Conditions and Participants

**Figure 3.**
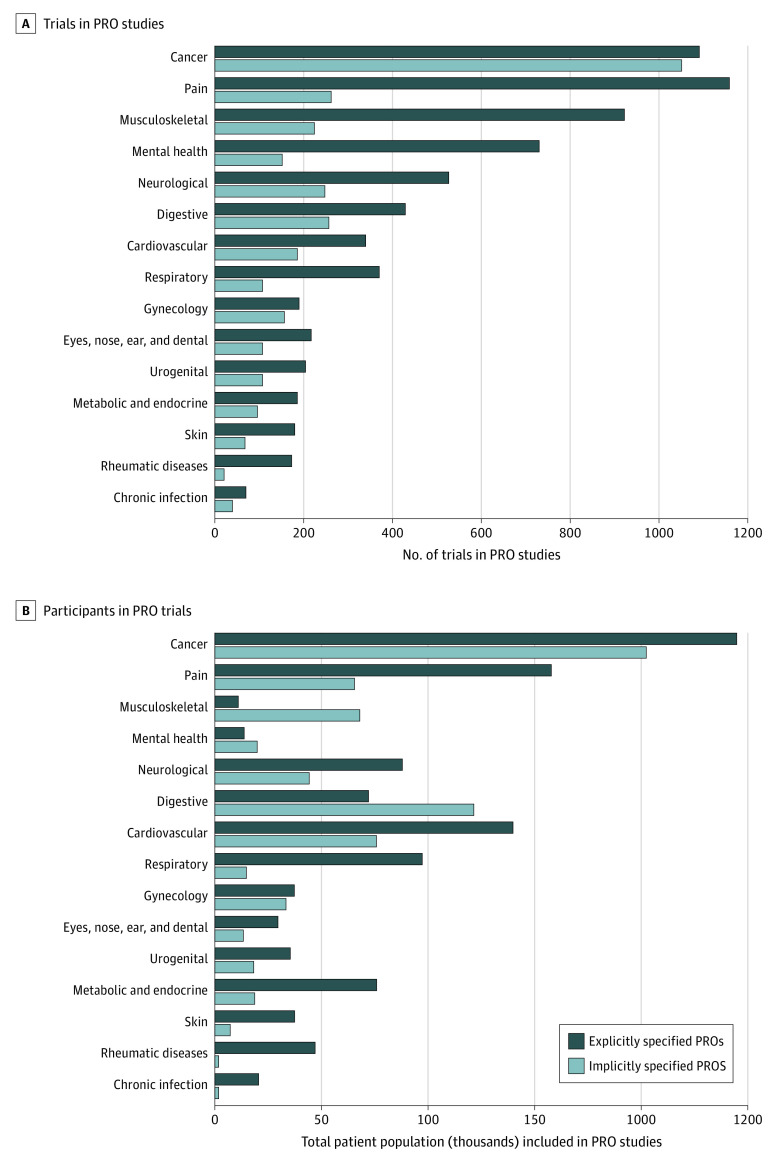
Number of Trials and Participants With Explicitly and Implicitly Specified Patient-Reported Outcomes (PROs)

During the study period, the number of clinical trials registered in China has continuously increased, with an annual growth rate of approximately 30%, and parallel growth was found for trials including PRO measurements. In the 6915 trials that listed specific PRO instruments, pain (16.8%), cancer (15.6%), musculoskeletal symptoms (13.3%), mental health conditions (10.6%), and neurologic diseases (7.6%) were the top 5 conditions for which PRO measures were considered as outcomes (Figure 3). Although the instruments used varied by disease type, visual analog scale (VAS), 36-item Short-Form Health Questionnaire (SF-36), and Hamilton Depression Scale were the most frequently used PRO tools in these trials (eTable 2 in the Supplement).

The number of conditions and participants in trials that included PRO measurements is shown in Figure 3. In the 10 093 trials that included PRO measures, cancer (21.2%), pain (14.1%), and musculoskeletal diseases (11.4%) were the most common conditions considered, followed by mental health (8.7%), neurologic (7.7%), digestive (6.8%), and cardiovascular (5.2%) conditions. Respiratory; gynecologic; ophthalmologic, nasal, auditory, and dental; and urogenital conditions accounted for 3% to 5% of these trials. Metabolic and endocrine conditions (2.8%), skin diseases (2.5%), rheumatic diseases (1.9%), and chronic infections (1.1%) were the least common conditions studied.

Of the 2 214 038 participants in trials that included PRO data, 20.2% (n = 447 413) were diagnosed with cancer, 10.1% (n = 223 482) were experiencing chronic pain, and 9.7% (n = 215 862) had cardiovascular conditions. Participants with digestive (193 740 [8.8%]), musculoskeletal (178 619 [8.1%]), mental health (157 776 [7.1%]), neurologic (132 393 [6.0%]), and respiratory (112 136 [5.1%]) conditions were also well represented (>10 000 participants in all cases). More than 50 000 participants in these trials had metabolic and endocrine (94 853 [4.3%]), gynecologic (70 919 [3.2%]), and urogenital (53 912 [2.4%]) conditions, and less than 50 000 participants in this group had rheumatic diseases (48 953 [2.2%]); skin conditions (44 867 [2.0%]); ophthalmologic, nasal, auditory, and dental conditions (43 104 [1.9%]); and chronic infections (39 598 [1.8%]).

### PRO Instruments Used in Clinical Trials

To evaluate the specific PRO instruments used in the 6915 trials that assessed patients’ subjective feelings in outcomes and explicitly mentioned the PRO collection tools, we categorized similar target diseases into 15 conditions (Table 2). The VAS and numeric rating scale were the most frequently used PRO scales in trials focused on a pain condition. For musculoskeletal diseases, most trials also used the VAS to assess the outcomes, followed by the Western Ontario and McMaster Universities Osteoarthritis index, patient quality of life, and SF-36. The Hamilton Depression Scale was used in 41.0% of mental health–related trials and in 13.8% of neurologic condition trials to assess depression. However, trials for mental health conditions more frequently focused on scales measuring insomnia with the Pittsburgh Sleep Quality Index and anxiety with the 14-item Hamilton Anxiety Scale, whereas neurologic trials seemed to largely focus on the VAS (16.5%) and European Quality-of-Life-5 Dimensions scale. Trials focused on digestive conditions tended to use more-specific instruments, such as the Irritable Bowel Syndrome Symptom Severity scale, Irritable Bowel Syndrome Quality-of-Life Questionnaire Scores, and Inflammatory Bowel Disease Questionnaire, along with the VAS and SF-36. Trials for respiratory conditions also used more condition-specific scales, including the St George’s Respiratory Questionnaire total scores, modified Medical Research Council dyspnea scale scores, and Chronic Obstructive Pulmonary Disease Assessment Test. Thus, for the 6915 trials that included PROs, the VAS (34.7%), SF-36 (8.8%), Hamilton Depression Scale (6.6%), Pittsburgh Sleep Quality Index (5.3%), and numeric rating scale (4.9%) were the top 5 tools used.

**Table 2.** Frequency of the Use of PRO Tools by Condition

Conditions	Proportion, No. (%)	PRO instruments											
		Name	No./total No. (%)	Name	No./total No. (%)	Name	No./total No. (%)	Name	No./total No. (%)	Name	No./total No. (%)	Name	No./total No. (%)
Total No.	6915												
Pain	1159 (16.8)	VAS	773/1159 (66.7)	NRS	190/1159 (16.4)	SF-36	75/1159 (6.5)	McGill Pain Questionnaire	39/1159 (3.4)	ODI	28/1159 (2.4)	SAQ	29/1159 (2.5)
Cancer	1080 (15.6)	QLQ-C30	308/1080 (28.5)	VAS	288/1080 (26.7)	EQ-5D	75/1080 (6.9)	NRS	53/1080 (4.9)	SF-36	22/1080 (2.0)	FACT-L	18/1080 (1.7)
Musculoskeletal	922 (13.3)	VAS	604/922 (65.5)	WOMAC	178/922 (19.3)	SF-36	159/922 (17.3)	ODI	52/922 (5.6)	SF-12	48/922 (5.2)	SF-MPQ	26/922 (2.8)
Mental health	730 (10.6)	HAMD	299/730 (41.0)	PSQI	191/730 (26.2)	HAMA	175/730 (24.0)	BDI/BAI	54/730 (7.4)	PHQ-9	35/730 (4.8)	SF-36	33/730 (4.5)
Neurological	527 (7.6)	VAS	87/527 (16.5)	HAMD	73/527 (13.9)	EQ-5D	48/527 (9.1)	PDQ-39	48/527 (9.1)	SF-36	32/527 (6.7)	PSQI	23/527 (4.4)
Digestive	429 (6.2)	VAS	132/429 (30.8)	SF-36	46/429 (10.7)	IBS-SSS	34/429 (7.9)	IBS-QOL	31/429 (7.2)	IBDQ	30/429 (7.0)	HAMD	26/429 (6.1)
Respiratory	370 (5.4)	SGRQ	90/370 (24.3)	mMRC	83/370 (22.4)	CAT	64/370 (17.3)	AQLQ	44/370 (11.9)	ACT	22/370 (6.0)	LCQ	19/370 (5.4)
Cardiovascular	340 (4.9)	SAQ	69/340 (20.3)	SF-36	56/340 (16.5)	MLFHQ	33/340 (9.7)	VAS	25/340 (7.4)	HAMD	14/340 (4.1)	EQ-5D	12/340 (3.4)
Ophthalmologic, nasal, auditory, and dental	217 (3.1)	VAS	114/217 (52.5)	RQLQ	22/217 (10.1)	NEI-VFQ-25	12/217 (5.5)	THI	11/217 (5.1)	OSDI	8/217 (3.7)	SPEED	7/217 (3.2)
Urogenital	204 (3.0)	VAS	46/204 (22.6)	IIEF	35/204 (17.2)	IPSS	35/204 (17.2)	ICIQ-SF	29/204 (14.2)	OAB-q SF	21/204 (10.3)	SF-36	14/204 (6.9)
Gynecology	190 (2.8)	VAS	67/190 (35.3)	SDA	22/190 (11.6)	EPDS	14/190 (7.4)	GCS	11/190 (5.8)	Men QOL	10/190 (5.3)	PFIQ	8/190 (4.2)
Metabolic and endocrine	186 (2.7)	VAS	39/186 (21.0)	SDACA	31/186 (16.7)	SF-36	17/186 (9.1)	SDS	12/186 (6.5)	HAMD	4/186 (2.2)	WHO-QOL	4/186 (2.2)
Skin	180 (2.6)	DLQI	84/180 (46.7)	VAS	76/180 (42.2)	NRS	15/180 (8.3)	Skindex16	10/180 (5.6)	HADS	6/180 (3.3)	HAMA	3/180 (1.7)
Rheumatic diseases	173 (2.5)	HAQ	44/173 (25.4)	DAS-28	30/173 (17.3)	SF-36	21/173 (12.1)	BASFI	29/173 (16.8)	BASDAI	29/173 (16.8)	ESSPRI	3/173 (1.7)
Chronic infection	70 (1.0)	WHOQOL-HIV	14/70 (20.0)	SF-36	10/70 (14.3)	HAMD	6/70 (8.6)	PSQI	6/70 (8.6)	VAS	6/70 (8.6)	EQ-5D	6/70 (8.6)

Abbreviations: ACT, Asthma Control Test; AQLQ, Asthma Quality of Life Questionnaire; BASDAI, Bath Ankylosing Spondylitis Disease Activity Index; BDI/BAI, Beck Depression Inventory-II; CAT, COPD Assessment Test; DAS-28, Disease Activity Score-28; DLQI, Dermatology Life Quality Index; EPDS, Edinburgh Postnatal Depression Scale; EQ-5D, European Quality of Life-5 Dimensions; ESSPRI, EULAR Sjogren's Syndrome Patient Reported Index; FACT-L, Functional Assessment of Cancer Therapy–Lung; GCS, Standard Greene Climacteric Scale; HADS, Hospital Anxiety Depression Scale; HAMA, 14-item Hamilton Anxiety Rating Scale; HAMD, Hamilton Depression Scale; HAQ, Health Assessment Questionnaire; IBDQ, Inflammatory Bowel Disease Questionnaire; IBS-QOL, Irritable Bowel Syndrome assessed by Quality of Life Questionnaire Scores; IBS-SSS, Irritable Bowel Syndrome Symptom Severity; ICIQ-SF, International Consultation on Incontinence Questionnaire–Short Form; IIEF, International Index of Erectile Function; IPSS, International Prostate Symptom Score; LCQ, Leicester Questionnaire; Men QOL, Menopause-Specific Quality of Life; MLFHQ, Minnesota Living With Heart Failure Questionnaire; mMRC, Modified Medical Research Council; NEI-VFQ-25, National Eye Institute Visual Functioning Questionnaire–25; NRS, numeric rating scale; OAB-q SF, Overactive Bladder Questionnaire Short Form; ODI, Oswestry Disability Index; OSDI, Ocular Surface Disease Index; PDQ-39, 39-item Parkinson Disease Questionnaire; PFIQ, PFIQ Urinary Impact Questionnaire; PHQ-9, Patient Health Questionnaire-9; PRO, patient-reported outcome; PSQI, Pittsburgh Sleep Quality Index; QLQ-C30, Quality of Life Questionnaire–Core 30; RQLQ, Rhinoconjunctivitis Quality of Life Questionnaire; SAQ, Seattle Angina Questionnaire; SDACA, Chinese version of Diabetes Self-Care Activities; SDS, Self-rating Depression Scale; SF-12, 12-item Short-Form Health Survey; SF-36, Short-Form 36-item Health Survey; SF-MPQ, Short-Form of McGill Pain Questionnaire; SGRQ, St George's Respiratory Questionnaire; Skindex16, Skindex-16 dermatologic survey; SPEED, Standard Patient Evaluation Eye Dryness; THI, Tinnitus Handicap Inventory; VAS, visual analog scale; WHOQOL-HIV, World Health Organization Quality of Life HIV instrument; WHO-QOL, World Health Organization Quality of Life-Lite-Clinical Trials Version; WOMAC, The Western Ontario and McMaster Universities Osteoarthritis Index.

## Discussion

This cross-sectional study evaluated the usefulness of PRO measures in randomized clinical trials conducted in China and quantitatively analyzed their characteristics. We found that 20.3% of the trials involving 4.7% of the total participants used PRO tools to assess the subjective experiences of patients. These data indicate the outcomes in the registration information of clinical trials. In fact, researchers found that PROs were frequently inadequate^20,21,22^ when protocols were reviewed in a rigorous scientific manner (Consolidated Standards of Reporting Trials or Standard Protocol Items: Recommendations for Interventional Trials–PRO Extension^24^). In our study, if we were successful in considering the protocols of these 20.3% of trials rigorously, then the reported PRO outcomes would have been lower than this percentage. We believe that the remaining 80% of the trials missed the opportunity to collect patients’ subjective evaluations. Clinical trials must highly prioritize patient benefit, and valuing patient opinion is important in making our trials more ethical. Although, to our knowledge, there are no similar studies for comparison, we suggest that it would be useful for Chinese researchers to recognize the importance of PROs and standardize the PRO collection process for clinical trials.

It is not surprising that cancer trials more commonly included PRO items; there are many innovative chemotherapy drugs that have emerged on the market and patient quality of life is one of the major research topics in the antitumor drug development field. PROs are increasingly being used in clinical practice; patient's opinions need to be considered not only in clinical trials, but also in routine health care. Some researchers believe that a patient-reported symptom should be included as a part of high-quality cancer care.^25^ The Chinese authorities released Guidelines for the Application of Patient-Reported Outcomes in Drug Clinical Research (Draft) in 2021 aimed at increasing the focus on PROs,^26^ which may contribute to improving the quality of clinical trials conducted in China.

We found that more than one-third of the trials that used PRO measures chose VAS as the main assessment tool. VAS is used for quick and easy access to data. Extensive use of VAS indicates that people are aware of PROs, which will encourage the development of more effective and reliable PRO tools. The necessity to develop suitable PRO tools for specific groups is widely acknowledged.^13,14,27,28^ Current high-quality randomized clinical trials are using PROs as end points.^8,29,30^ In our study, we did not identify any trial that used a self-developed PRO tool during the study years. However, a positive sign is that some researchers have begun to focus on the development and evaluation of new PRO scales.^9,31^ Some Chinese researchers have also recognized the great potential of PROs and have begun to independently design novel PRO instruments^32,33,34,35^ and apply them in clinical practice.^36,37^

The findings of this survey are concordant with our hypothesis that phase 3 and phase 4 trials typically involve more PRO measures. PRO items were included in 12.9% of phase 4 clinical trials, suggesting that researchers may be willing to pay more attention to patients’ feelings when the trial is not aimed only at marketing. However, the omission of PRO content among trial outcomes in the early stages may reduce the value of patient participation in trials.^27^ We found that the request for indication of the phase on the databases was marked as other or not applicable by the registrant for more than 30% of the trials. Therefore, our statistics are probably in line with the actual information. Further attention should be given to registering clinical trials for improved ethics compliance.

In this study, we also found that there were obvious regional differences in PRO use. In the eastern, northern, and southern regions of China, PRO measurements were much more frequently adopted, although trials conducted in remote areas may need to be simplified for logistic reasons. PROs cannot only provide references for more ambitious trials but also serve as the vital basis for labeling claims on noninnovative drug trials, such as bioequivalence studies.^38,39,40^ Even less-developed regions or countries may benefit from adopting PROs for clinical trials.

Clinical trials conducted in China may be important in terms of PRO assessment, given that PRO data collection and management are appropriately performed. Thus, China is in the position to establish its PRO system and design and enrich its PRO instruments based on data from the current situation and past years.

### Limitations

This study has limitations. We excluded trials in children, which may have caused a bias in the results and excluded special cases, given the substantial amount of work emerging on pediatric PROs.^41,42^ During data extraction, we found that some registrants did not rigorously supply the details of the trial; for example, approximately 2010 registered trials were still marked as being in recruitment status, which was not the case. Therefore, we did not consider recruitment status, which may have skewed the sample size. Although the World Health Organization has mandated registration for all clinical trials, we are concerned that there were omissions in some cases. We also noticed that it was not always clear whether PRO assessments were performed because of insufficient or unclear reporting of methods. Overall, 3178 trials were marked as having implicitly specified PROs. To limit misclassification, at least 3 rounds of review by 2 senior researchers were conducted independently.

## Conclusions

This cross-sectional study found that in China, patients’ experiences and expectations with treatment in clinical trials require more attention. Patient-reported responses to treatment can provide valuable clinical evidence. Documentation of patients’ subjective feelings and details on quality of life in the context of chronic diseases, such as cardiovascular diseases, respiratory diseases, rheumatic diseases, skin conditions, and digestive conditions, is necessary. Further efforts in the development of PRO scales that can be adapted to the Chinese population would be beneficial.

## Notes

Supplement. **eMethods.** Search Strategy **eFigure.** The Percentage of Trials (Explicitly Specified PROs and Implicitly Specified PROs) in Each Province **eTable 1.** Classification of Specific Diseases **eTable 2.** PRO Tests Used Most Frequently
