# Supplementary material for: Application of Patient-Reported Outcome Measurements in Clinical Trials in China
Source: JAMA Netw Open. 2022 May 11;5(5):e2211644. doi: 10.1001/jamanetworkopen.2022.11644 (PMC9096600; doi:10.1001/jamanetworkopen.2022.11644)
Supplement: Supplement. — eMethods. Search Strategy eFigure. The Percentage of Trials (Explicitly Specified PROs and Implicitly Specified PROs) in Each Province eTable 1. Classification of Specific Diseases eTable 2. PRO Tests Used Most Frequently [file jamanetwopen-e2211644-s001.pdf]

Supplementary Online Content

Zhou H, Yao M, Gu X, et al. Application of patient-reported outcome measurements in clinical trials in China. *JAMA Netw Open*. 2022;5(5):e2211644. doi:10.1001/jamanetworkopen.2022.11644

eMethods. Search Strategy

eFigure. The Percentage of Trials (Explicitly Specified PROs and Implicitly Specified PROs) in Each Province

eTable 1. Classification of Specific Diseases

eTable 2. PRO Tests Used Most Frequently

This supplementary material has been provided by the authors to give readers additional information about their work.

© 2022 Zhou H et al. *JAMA Network Open.*

eMethods. Search Strategy

1. **Search strategy for www.chictr.org.cn**

“Study type” = Interventional Study

“Study phase” = 1, 2, 3, 4, 1-2, 2-3, 0, 4, N/A,

“First Posted” = “01/01/2010” To “31/12/2020”

“sex” = Male, Female, Both

“Country” = China

2. **Search strategy for ClinicalTrials.gov**

“Study type” = Interventional Studies (Clinical Trials)

“Phase” = Early phase 1, Phase 2, Phase 3, Phase 4, Not Applicable

“First Posted” = “01/01/2010” To “31/12/2020”

“Sex” = Studies with Male Participants, Studies with Female Participants, All

“Country” = China

© 2022 Zhou H et al. *JAMA Network Open.*

e Figure The Percentage of Trials (Explicitly Specified PROs and Implicitly Specified PROs) in Each Province.

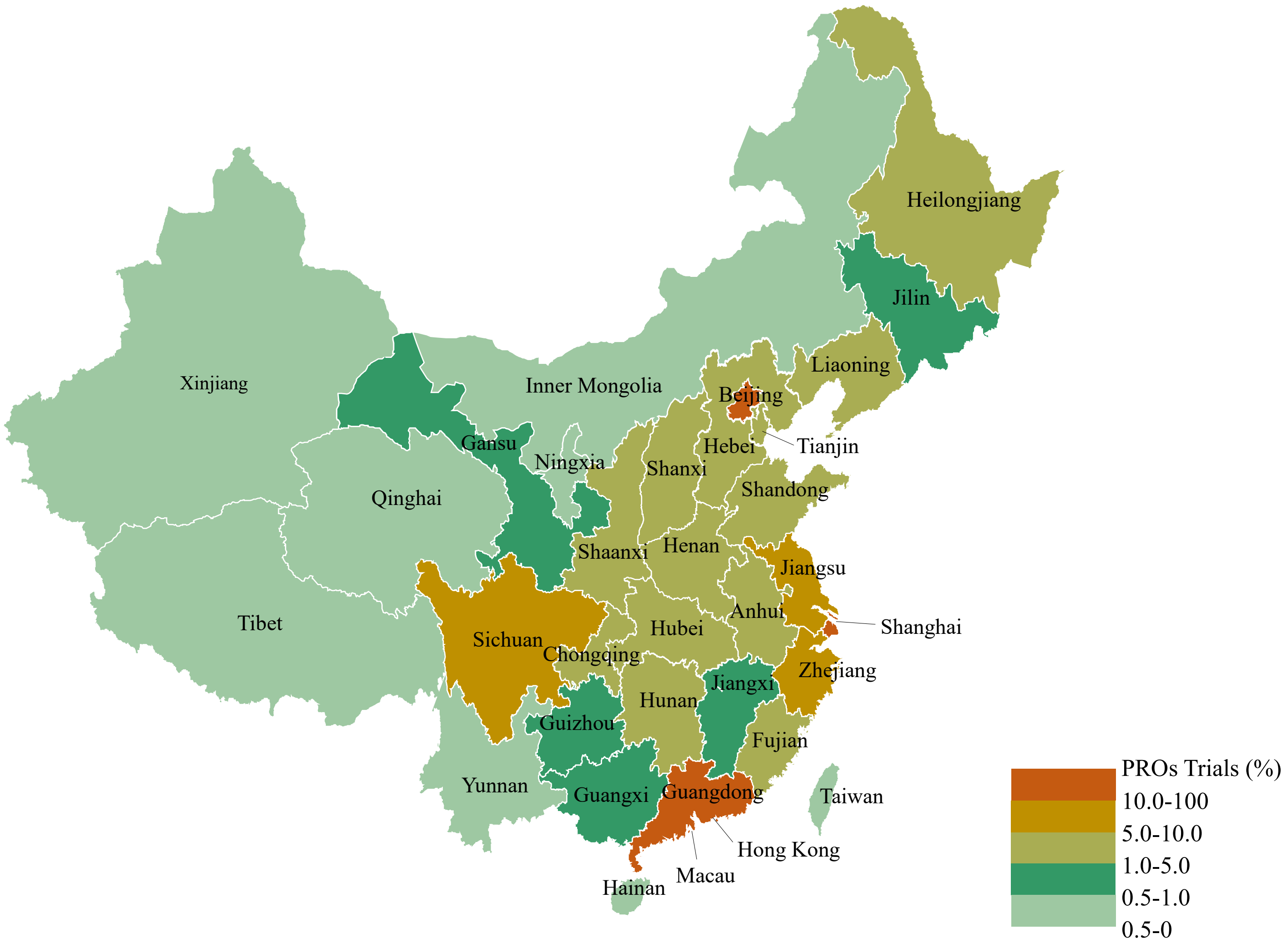

© 2022 Zhou H et al. *JAMA Network Open.*

eTable 1. Classification of Specific Diseases

	Classification	Specific Diseases				
1	Cancer Set	Brain	Nasopharynx	Oral	Esophagus	Lung
		Rectum	Duodenum	Small Intestine	Large Intestine	Cervix
		Gallbladder	Bile Duct	Skin	Uterus	Lymph
		Prostate	Tongue	Blood	Head and Neck	Pituitarium
		Stomach	Pancreas	Liver	Bladder	Peritoneum
		Oophoron	Breast	Thyroid	Sigmoideum	Larynx
		Spine	Bone	Thymus	Kidney	Urothelium
2	Pain	Pain	Back Pain	Neck Pain	Chronic Migraine	Chronic Pelvic Pain
		Pain Management	Perinatal Analgesia	Labor Analgesia	Myofascial Pain	Non-Cancer Pain
		Phantom Limb Pain	Injection Pain	Trigeminal Neuralgia	Fibromyalgia	Perioperative Analgesia
		Chronic Regional Pain Syndrome	Hyperpathia			

© 2022 Zhou H et al. *JAMA Network Open.*

3	Musculoskeletal	Meniscus Injury	Cruciate Ligament Injury	Fracture	Patellar Dislocation	Patellar Tenosynovitis
		Frozen Shoulder	Joint Replacement	Joint Dislocation	Achilles Tendon Rupture	Achilles Tendon Contracture
		Acute Achilles Tendon Rupture	Anterior Cruciate Ligament Injury	Spinal Cord Injury	Cervical Spondylosis	Pathological Fractures of the Spine
		Rotator Cuff Degeneration	Rotator Cuff Injury	Frozen Shoulder	Calcium Pyrophosphate Deposition Disease	Abnormal Cervical Spine Physiological Curvature
		Anterior Cruciate Ligament Reconstruction	Scoliosis	Flexor Tendon Stenosis Tenosynovitis	Aseptic Loosening of Artificial Joints	Aseptic Loosening of Total Hip Arthroplasty
		Occupation-related Musculoskeletal Disorders	Plantar Fasciitis	Stiff Elbow Joint	Shoulder-hand Syndrome	Meniscus in Knee Osteoarthritis
		Traumatic and Limb Defects	Cervical Spondylosis	Periarthritis of the Shoulder	Shoulder-hand Syndrome	Decreased Bone Mass
		Ankle Arthritis	Myofasciitis	Myelodysplastic Syndrome	Anterior Cruciate Ligament Injury	Orthopedic Incision
		Traumatology and Orthopedics	Post-traumatic Elbow Stiffness	Ischemic Necrosis of the Femoral Head	Osteoarthritis	Subacromial Bursitis

© 2022 Zhou H et al. *JAMA Network Open.*

		Biceps Tendon Injury of Shoulder Joint	Severe Comminuted Fractures	Sarcopenia	Joint Adhesions	Enlarged Jaw Angle
		Ankle Sprains	Ulnar Impingement Syndrome	Hemorrhagic Synovitis	Degenerative Diseases of the Cervical Spine	Osteoradionecrosis of the Jaws
		Functional Ankle Instability	Plantar Fasciitis	Femoral Head Necrosis	Soft-tissue Contusions	Carpal Tunnel Syndrome
		Spinal Instability	Spinal Deformity	Dysplasia of the Hip	Fracture of the Femur	Femoral Head Replacement
		Lumbar Degenerative Diseases	Articular Cartilage Defects	Articular Cartilage Degeneration	Orthopedic Anesthesia	Orthopedic Spine Surgery
		Tuberculosis of the Spine	Anterior Cruciate Ligament Reconstruction with Autologous Hamstring	Osteoporosis	Ectropion	Orthopedic Skin Incision
		Subacromial Impingement Syndrome	Temporomandibular Joint Disorders	Temporomandibular Joint Disorder	Tennis Elbow	Lumbar Disc Herniation
		Soft-Tissue Injury of the Ankle Joint	Myofascial Pain Syndrome	Lumbar Spinal Stenosis		

© 2022 Zhou H et al. *JAMA Network Open.*

4	Mental health	Anxiety Disorders	Online Game Barriers	Opioid Withdrawal Syndrome	Opioid Dependence	Post-traumatic Stress Disorder
		Heroin Addiction	Obsessive Compulsive Disorder	Panic Disorder	Schizophrenia	Paranoia
		Major Depressive Disorder	Suicidal Ideation	Methamphetamine Addiction	Methamphetamine Addiction	Insomnia
		Addiction	Autism	Emotional Disorders	Nervousness Syndrome	Drug Abuse
		Bad State of Mind	Postnatal Anxiety	Postnatal Depression	Hysteria	Cognitive Impairment
		Depression	Drowsiness	Major Depressive Disorder	Drug Addiction	Social Anxiety
		Alcohol Withdrawal	Alcohol Dependence	Geriatric Depression	Cigarette Addiction	Drug Addiction
		Sleep Disorders	Perinatal Depression	Perioperative Anxiety	Social Fear	Bipolar Disorder
		Delirium				

© 2022 Zhou H et al. *JAMA Network Open.*

5	Neurological	Alzheimer's Disease	Alzheimer's Disease	Chemotherapy-induced Peripheral Neurotoxicity	Parkinson's Disease	Restless Leg Syndrome
		Cauda Equina Injury	Tourette’s and Chronic Disorders	Meniere's Disease	Facial Muscle Spasm	Facial nerve Palsy
		Smoking-Related Disease	Central Nervous System Diseases	Stroke	Giddiness	Neurocognitive Disorders
		Huntington's Disease	Acute Ischemic Cerebrovascular Disease	Idiopathic Facial Neuritis	Vascular Dementia	Burning Mouth Syndrome
		Dementia	Nerve Damage	Epilepsy	Neurosurgery	Idiopathic Tremor
		Hirayama Disease	Cognitive Dysfunction	Aphasia	Multiple Tourette Syndrome	Post-stroke Sequelae
		Peripheral Nerve Injury	Autonomic Disorders	Peripheral Neuropathy	Glossopharyngeal Neuralgia	

© 2022 Zhou H et al. *JAMA Network Open.*

6	Digestive	Constipation	Intestinal Dysfunction	Fatty Liver	Cholecystitis	Benign Gallbladder Disease
		Irritable Bowel Syndrome	Colonoscopy	Gallbladder Stones	Gallbladder Polyps	Gallbladder Surgery
		Malignant Obstruction of the Biliary Tract	Upper Gastrointestinal Tract Diseases	Intractable Eruption	Delayed Gastric Emptying Disorder	Drug-related Liver Injury
		Pancreatic Insufficiency	Resistance Jaundice	Acute Perforation of the Upper Gastrointestinal tract	Acute Pancreatitis	Crohn's Disease
		Functional Indigestion	Acute Appendicitis	Anal fistula	Perianal Diseases	Anorectal Surgery
		Gastrectomy	Cirrhosis of the Liver	Liver Transplant	Anal Fissure	Gastrointestinal Surgery
		Gastroesophageal Reflux Disease	Complex Abdominal Wall Defects	Inguinal Hernia	Intestinal Injury	Esophageal Hiatal Hernia
		Aphthous Ulcer	Complex Anal Fistula	Colorectal Surgery	Diarrhea	Pancreatitis
		Ulcerative Colitis	Non-atrophic Gastritis	Gastrointestinal Disorders	Gastroparesis	Gastric Mucosal Injury

© 2022 Zhou H et al. *JAMA Network Open.*

		Helicobacter Pylori Infection	Intestinal Failure	Lean Functional Dyspepsia	Belching	Pharyngeal Reflux Syndrome
7	Cardiovascular	Heart Valve Disease	Stable Angina Pectoris	Acute Myocardial Infarction	Acute Myocardial Infarction	Atrial Tachycardia
		Coronary Artery Bypass Grafting	Angina Pectoris	Acute ST-segment Elevation Myocardial Infarction	Chronic Systolic Heart Failure	Cardiovascular and Cerebrovascular Events
		Percutaneous Transluminal Coronary Intervention	Arrhythmia	Carotid Artery Unstable Plaque	Chronic Stable Coronary Artery Disease	Post-operative Cardiac Surgery
		Pulmonary Arterial Hypertension	Acute Coronary Syndrome	Venous Thromboembolism	Embolic Pulmonary Hypertension	Cardiac Arrest
		Obstructive Hypertrophic Cardiomyopathy	Acute Decompensated Heart Failure	Chronic Congestive Heart Failure	Ischemic Heart Disease	Hypertension
		Cardiovascular Metabolic Syndrome	Elevated Blood Cholesterol	Cardiovascular Disease	Dyslipidemia	People at Risk for Cardiovascular Disease

© 2022 Zhou H et al. *JAMA Network Open.*

		Aortic Coarctation	Heart Surgery Related Diseases	Aortic Valve Insufficiency	Lower Extremity Deep Vein Thrombosis	Arrhythmia
		Aortic Valve Stenosis	Slow Arrhythmias	New Onset of Postoperative Atrial Fibrillation	Mitral Valve Lesions	Phlebitis
		Sinus Node Syndrome	After Mechanical Valve Replacement	Lymphoedema	Atrial Fibrillation	Varicose Veins
		Atherosclerosis	Aortic Coarctation	Premature Ventricular Contractions	Thrombo-occlusive Vasculitis	Cardiac Neurosis
		Venous Ulcers of the Lower Extremities	Heart Failure	Hypotension	Severe Lower Limb Ischemic Disease	Ischemic Cardiomyopathy
		Congenital Heart Disease	Peripheral Artery Disease	Atrial Fibrillation	Lower Extremity Atherosclerosis Occlusive Disease	Myocardial Infarction

© 2022 Zhou H et al. *JAMA Network Open.*

8	Respiratory	Pulmonary Aspergillosis	Pneumoconiosis	Asthma	Pulmonary Nodules	Lung Lobectomy
		Pneumonia	Bronchial Asthma in Adults	Bronchial Dilation	Lung Atrophy	Cough
		Sleep Apnea Syndrome	Lower Respiratory Tract Infections	COVID-19	Chest Trauma	Chest and Lung Diseases
		Silicosis	Cold and Flu	Community-acquired Pneumonia	Airway Hypersecretion	Idiopathic Pulmonary Fibrosis
		Allergic Respiratory Tract Inflammation	Bronchitis	Acute Pharyngitis	Pneumothorax	Smoke Inhalation Lung Injury
		Acute Respiratory Failure	Upper Respiratory Tract Infection	Tonsillitis	Connective Tissue Disease-associated Interstitial Lung Disease	Lung Transplant
		Bronchoscopy	Pulmonary Alveoli	Interstitial Lung Disease	Chronic Obstructive Pulmonary Disease	Influenza A
		Geriatric Severe Pneumonia	Chronic Respiratory Diseases			

© 2022 Zhou H et al. *JAMA Network Open.*

9	Gynecology	Infertility	Trichomonas Vaginalis	Recurrent Miscarriage	Cervical Insufficiency	Perineal Fissure
		Insufficient Milk Supply	Polycystic Ovary Syndrome	Laparoscopic Gynecological Surgery	Uterine Adhesions	Acute Mastitis
		Postnatal Rehabilitation	Childbirth	Menopausal Syndrome	Itching in the Perineum	Premenstrual Syndrome
		Perimenopausal Depression	Abnormal Uterine Bleeding	Menstrual Disease	Early-onset Ovarian Insufficiency	Premature Ovarian Failure
		Nausea and Vomiting in Pregnancy	Perimenopausal Hot Flashes	Mastopathy	Perimenopausal Panic Disorder	White Lesions of the Vulva
		Hysteroscopic Surgery under Intravenous Anesthesia	Menopausal Genitourinary Syndrome	Chronic Cervicitis	Female Sexual Dysfunction	Breast Surgery
		Pelvic Floor Organ Prolapse	Poor Healing of Cesarean Incision	Postpartum Urinary Incontinence	Induced Abortion	Cesarean Delivery
		Menopausal Transition	Menopausal Syndrome	Endometriosis	Mastitis during Lactation	Anesthesia for Caesarean Section
		Menopausal hypertension	Ovarian Hyporesponsiveness	Non-lactating Mastitis	Perimenopausal Anxiety	Vaginal Candidiasis

© 2022 Zhou H et al. *JAMA Network Open.*

		Menopausal Mood Disorders	Decreased Ovarian Function	Pelvic Floor Dysfunction	Breast Pain	Mastopexy
		Pregnancy	Labor Pains	Childbirth Experience	Uterine Fibroid Removal	Fear of Childbirth
		Premature Miscarriage	Obesity during Pregnancy	Painless Abortion	Menstrual Pain	
10	Eyes, Nose, Ear and Dental	Cataracts	Deviated Nasal Septum	Tinnitus	Dental Fluorosis	Allergic rhinitis
		Narrowing of the nasal vestibular area	Sinusitis	Deafness	Mouth ulcers	Conjunctivitis
		Nasal polyps	Exfoliative Lip Infection	Mucositis of the Oral Cavity	Dry Eye	Acute Pharyngitis
		Dentin Sensitivity	Missing Teeth	Dental and Maxillofacial Malformations	Pulpitis	Gum Recession
		Obstructive teeth	Vitreoretinal Diseases	Vocal fold polyp	Oral Candidiasis	Lacrimal Obstructive Disease
		Blepharospasm dysfunction	Craniomaxillofacial Malocclusion	Chronic Subjective Tinnitus	Periodontitis	Refractory Amblyopia
		Nearsightedness	Chronic Hypertrophic Rhinitis	Sudden Deafness	Endodontic Infection	Granulomatous Labyrinthitis

© 2022 Zhou H et al. *JAMA Network Open.*

		Oral Erosive Lichen Planus	Suppurative Otitis Media	Temporomandibular Joint Disorder Syndrome	Occlusal Disorders	Sudden Deafness
		Glaucoma	Visual Fatigue	Refractive Eye Strain	Retinal Vein Obstruction	
11	Urogenital	Overactive Bladder Syndrome	Erectile Dysfunction	Bladder Irritation	Urge Incontinence	Prostatic Hyperplasia
		Hypermobility of the Bladder	Sexual Renal Cysts	Stress Urinary Incontinence	Interstitial Cystitis	Prostatitis
		End-Stage Renal Failure	Acute Kidney Injury	Kidney Transplantation	Overcircumcision	Urinary Tract Stones
		Bladder Neck Contracture	Bladder Discomfort	Peritoneal Dialysis	Urinary Tract Infections	Urethroscopic Surgery
		Urinary Stones	Urethral Stricture	Renal Failure	Premature Ejaculation	Chronic Kidney Disease
		Male Hypogonadism	Uremia	Erectile Dysfunction	End-stage Renal Disease	Hemodialysis
		Acute Renal Colic	Female Overactive Bladder Syndrome	Prostate Electrosurgery		

© 2022 Zhou H et al. *JAMA Network Open.*

12	Metabolic and endocrine	Obesity	Metabolic Diseases	Laparoscopic Bariatric Surgery	Thyroidectomy	Goiter
		Diabetic Nephropathy	Diabetes and all its Complications	Thyroid-related Ophthalmopathy	Geriatric Malnutrition	Gestational Diabetes
		Hashimoto's Thyroiditis	Adrenocortical Suppression	Subacute Thyroiditis	Hyperparathyroidism	Frailty
		Androgenic Alopecia	Malnutrition	Adult growth Hormone Deficiency	Diabetes Mellitus	Gout
		Diabetes	Metabolic Syndrome	Thyroid Disorders		
13	Skin	Medicine related to Itchy Skin	Baldness	Flat Warts	Eczema	Melasma
		Vitiligo	Scarring	Acne	Dark Circles under the Eyes	Condyloma Acuminatum
		Atopic Dermatitis	Drug-related Rashes	Seborrheic Alopecia	Pemphigus Vulgaris	Skin Burns
		Neck Wrinkles	Psoriasis	Facial Laxity	Freckles	Wine Spots
		Periorbital Wrinkles	Urticaria	Itchy Skin	Decubitus Ulcers	Skin Trauma
		Actinic Keratosis	Ichthyosis	Tinea pedis	Skin Ulcers	Skin Defect with Implants
		Herpes Zoster	Radiation Skin Injury			

© 2022 Zhou H et al. *JAMA Network Open.*

14	Rheumatic Diseases	Rheumatoid Arthritis	Ankylosing Spondylitis	Lupus Nephritis	Raynaud's Phenomenon	Systemic Lupus Erythematosus
		Psoriatic Arthritis	Primary Sjögren's Syndrome			
15	Chronic Infection	AIDS	Herpes	Tuberculosis	Hepatitis C	Hepatitis B
		Sepsis				
16	Hematological	Myelodysplastic Syndromes	Primary Thrombocythemia	Thrombocytopenia	Hemophilia A	Idiopathic Thrombocytopenic Purpura
		Hematopoietic Stem Cell Transplantation	Severe Aplastic Anemia			
17	Other	Health People	Highland Reaction			

© 2022 Zhou H et al. *JAMA Network Open.*

eTable 2. PRO Tests Used Most Frequently

Test	Frequency of Use, No. (%)
VAS	2397 (34.7)
SF-36	606 (8.8)
HAMD	454 (6.6)
PSQI	369 (5.3)
NRS	336 (4.9)
QLQ-C30	312 (4.5)
HAMA	298 (4.3)
SDS	268 (3.9)
SAS	234 (3.4)
WOMAC	233 (3.4)
HADS	131 (1.9)
SF-12	127 (1.8)

HADS, Hospital Anxiety Depression Scale; HAMA, 14-item Hamilton Anxiety Rating Scale;
HAMD, Hamilton Depression Scale; NRS, numeric rating scale; PSQI, Pittsburgh Sleep Quality Index;
QLQ-C30, Quality of Life Questionnaire–Core 30; SAS, Self-rating Anxiety Scale; SDS, Self-rating Depression Scale;
SF-12, 12-item Short-Form Health Survey; SF-36, Short-Form 36-item Health Survey; VAS, visual analog scale;
WOMAC, The Western Ontario and McMaster Universities Osteoarthritis Index.

© 2022 Zhou H et al. *JAMA Network Open.*
